# Supplementary material for: Novel Model for Stomatal Conductance: Enhanced Accuracy Under Variable Irradiance and CO2 in C3 Plant Species
Source: Biology (Basel). 2025 Oct 27;14(11):1501. doi: 10.3390/biology14111501 (PMC12650297; doi:10.3390/biology14111501)
Supplement: Supplementary file 1 [file biology-14-01501-s001.zip › biology-3907863-supplementary.pdf]

Table S1 Definitions of the abbreviations

Abbreviation	Definition	Units
A_n	Net photosynthetic rate	μmol m^{-2} s^{-1}
A_n–C_i	CO_2-response curve of photosynthesis	dimensionless
A_n–I	Light-response curve of photosynthesis	dimensionless
A_{max}	Maximum net photosynthetic rate	μmol m^{-2} s^{-1}
C_a	Atmospheric CO_2 concentration	μmol mol^{-1}
C_i	Intercellular CO_2 concentration	μmol mol^{-1}
C_s	Leaf surface CO_2 concentration	μmol mol^{-1}
g_0	Residual stomatal conductance when A_n is zero	mol m^{-2} s^{-1}
g_1	Empirical slope	dimensionless
g_{sc}	Stomatal conductance to CO_2	mol m^{-2} s^{-1}
h_r	Relative humidity	dimensionless
I	Light intensity	μmol m^{-2} s^{-1}
I_{sat}	Saturation light intensity	μmol m^{-2} s^{-1}
J–C_i	CO_2 -response curves of electron transport rate	dimensionless
J–I	Light-response curves of electron transport rate	dimensionless
R_{day}	Day respiratory rate in light or with a constant irradiance and changing CO_2 concentration	μmol m^{-2} s^{-1}
Γ_*	Photorespiratory CO_2 compensation point in the absence of R_{day}	μmol $μmol^{-1}$
Γ	Photorespiratory CO_2 compensation point in the presence of R_{day}	μmol $μmol^{-1}$
λ	Critical water use efficiency	μmol mol^{-1}
